# Patient Comfort, Acceptance, and Tolerability of Virtual Reality (VR) Headsets with Real-Time Eye Tracking for Remote Visual Field Testing

**DOI:** 10.3390/jcm14093219

**Published:** 2025-05-06

**Authors:** Athena Lallouette, Kevin Gillmann

**Affiliations:** 1 Genève Ophtalmologie, CH-1205 Geneva, Switzerland; athena@geneve-ophtalmologie.ch; 2 University of London, Queen Mary, London WC1E 7HU, UK

**Keywords:** functional testing, visual field, perimetry, artificial intelligence, virtual reality, home testing

## Abstract

**Objectives**: Visual field (VF) testing is key to assessing functional loss in glaucoma. Despite its clinical value, traditional VF testing has a number of limitations, including its dependency on medical equipment and posturing requirements. The present study examines the acceptance, tolerability, and comfort of home-based VF testing using a virtual reality (VR) headset. **Method**: Healthy subjects were prospectively enrolled to undergo VF examination in a non-clinical setting using a commercially available stereoscopic VR headset and SORS (sequentially optimized reconstruction strategy) on the VisionOne platform. Subjects were supervised and wore their own spectacles within the headset. After the VR VF test, they were asked about their comfort, side effects, and readiness to repeat the examination at home. **Results**: Of the 12 subjects enrolled, 7 were female (58.3%) and 5 were male (41.7%). Mean age was 45 years (range: 30–68). While none of the subjects suffered from glaucoma, their medical histories included severe arthritis, refractive surgery, high myopia, amblyopia, and esotropia. The mean self-reported comfort score was 8.75 out of 10 (range: 8–10), with some subjects taking the test in the dorsal decubitus position. Eleven subjects (91.7%) considered the device to be easy to use, and 100% responded that they would be willing to repeat the test at home, of which 41.7% stated they would prefer to be supervised by a clinician. Overall, three subjects reported mild side effects, namely light asthenopia, epiphora, and periocular flushing. All side effects were mild and self-limited. The mean perceived duration of the test (187 s) correlated strongly with the mean actual duration (166 s; Pearson correlation coefficient r = 0.76, *p*-value = 0.007). In all, 58.3% of perceived durations were shorter than the actual test durations. Mean false negative and false positive responses were 3.75% and 4.7%, respectively. Central fixation recorded by real-time eye tracking was maintained on average 73.23% of the time and showed a strong correlation with false negative responses (r = 0.75; *p* = 0.026). **Conclusions**: While the present study did not examine the test algorithm itself, it suggests that home-based VF testing using a VR headset is well tolerated and accepted, with high levels of self-reported comfort and only mild side effects. While all subjects welcomed the opportunity to perform clinical tests from home, over a third expressed a preference for supervision. Real-time eye tracking correlated well with traditional reliability markers, suggesting potential clinical value.

## 1. Introduction

Automated perimetry or visual field testing (VF) is a non-invasive technique used to assess and monitor dysfunctions of the visual pathways and ophthalmologic pathologies. It has evolved to become the standard method used to assess visual function in glaucoma and a crucial tool to diagnose and evaluate disease progression alongside structural tests [1].

Standard perimetry assessment tests the patient’s ability to consciously detect light stimuli of varying intensities and sizes, over a calibrated illuminated background, in targeted visual field locations, typically within the central 30 degrees of the visual field. The threshold sensitivity of different locations of the retina is then assessed, provided that the visual central fixation is maintained throughout the test. The standardization of automated perimetric testing allows the comparison and statistical analysis of visual field defects.

Despite its unquestionable clinical value, traditional automated visual field testing has several limitations. These include its dependency on large medical equipment and postural requirements. The subjective nature and psychological elements of perimetric responses are associated with a long learning curve and high test–retest variability that may also complicate the assessment of the results [2]. Perimetry testing is known to induce anxiety in glaucoma patients due to their fear of not being able to answer correctly and test-induced fatigue, particularly in longer tests, which may further affect test reliability [3].

Visual field testing using a virtual reality (VR) headset may present potential benefits for patients in terms of comfort, with a potential positive impact on test reliability and adherence [4].

The present study examines the acceptance, tolerability, and comfort of visual field (VF) testing using a VR headset in a non-clinical setting. In doing so, it explores one aspect of the broader feasibility of shifting visual field assessment from clinical to non-clinical environments using portable VR technology.

## 2. Materials and Methods

This project was conducted as a service evaluation audit to explore the acceptability and potential benefits for patients of transitioning from traditional in-clinic visual field testing to the use of a VR-based CE-approved device in a non-clinical setting. As such, and in accordance with institutional policy, a review by a formal institutional review board was not required. The study adhered to the tenets of the Declaration of Helsinki.

**Figure 1:**
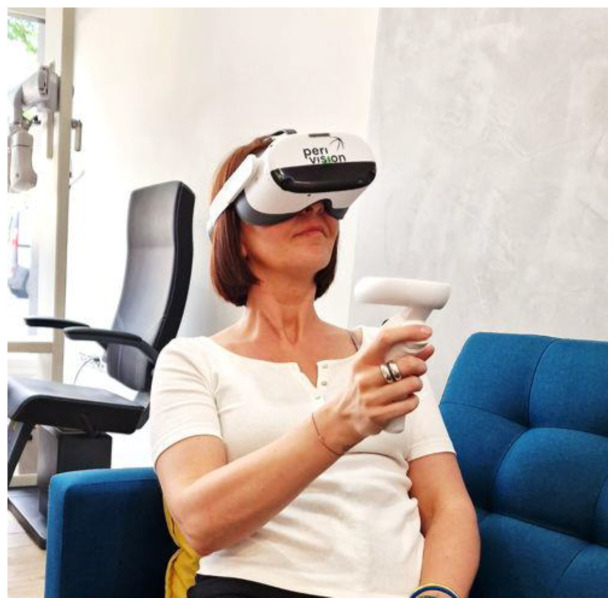
VR headset: Pico Neo 3 Pro.

Subjects were recruited prospectively from a primary care eye clinic (Genève Ophtalmologie, Geneva, Switzerland) by their usual care provider during routine eye care. All subjects underwent VF examination in a non-clinical setting using a commercially available stereoscopic VR headset, namely a Pico Neo 3 Pro (ByteDance, Beijing, China; Figure 1). The device includes a headset and a remote controller, weighing about 600 g, and is equipped with Fresnel lenses offering a total visual field of 98 degrees. The LCD (liquid crystal display, ByteDance, Beijing, China) screen has a 1830 × 1920 pixel resolution and a maximum brightness of 104 cd/m^2^. The headset features built-in real time eye-tracking functionalities, notably used to produce an eye-tracking score representing the proportion of time that the gaze was on target. The eye-tracking calibration takes place at the start of every VF test and is carried out monocularly, avoiding any issues with subjects who present altered binocular vision or heterotropia. In addition, traditional reliability indices, such as false positives and false negatives, are monitored in a manner comparable to conventional perimetry. During the test, the headset prompts the user with an audio message to correct any fixation loss for each individual eye. At the end of each test, the system automatically uploads the test results via Wi-Fi onto a web-based platform accessible to the clinical practitioner (VisionOne, PeriVision, Lausanne, Switzerland).

Both eyes were tested monocularly, with the patients’ own spectacles, where no eyepatch was necessary, as the VF device only displayed stimuli to the tested eye. The test followed a 24-2 pattern using a SORS (sequentially optimized reconstruction strategy; PeriVision, Lausanne, Switzerland) strategy, a glaucoma-trained artificial intelligence-based VF strategy developed by the University of Bern and the University Hospital of Bern, in Switzerland. SORS uses the learnt correlations between the retinotopic test locations to reconstruct untested locations, achieving high correlations with the clinical gold standard Dynamique Strategy while reducing the test duration [5]. The test stimuli varied in luminance between 10 and 110 cd/m^2^, with a standard Goldmann size of III.

The subjects were supervised and received the same instructions by the same certified orthoptist throughout this study. After VF testing, a semi-structured qualitative interview was conducted as well as a standardized survey based on a French version of the Client Satisfaction Questionnaire-8 (CSQ-8) [6,7], where subjects were asked about their comfort using a 10-point Likert scale, with 10 representing the most positive score. Spontaneous qualitative feedback, self-reported side effects, ease of use, readiness to repeat the test at home with or without medical supervision, and perceived duration for each test were also recorded. Qualitative comments were subjectively categorized as positive, neutral, or negative by the authors (AL). All questionnaires and interviews were conducted in French, and no external software or codebook were used in their analysis.

Demographic information was also collected, including the subjects’ age, gender, race and ophthalmologic history. Some test data were also collected from the VisionOne platform, including the duration of the tests, eye-tracking score, false positives (FPs), false-negatives (FNs), and fixation losses (FLs). Descriptive statistics were presented using percentages, ranges, and standard deviations, and correlations were analyzed between perceived test duration, actual duration, as well as eye-tracking and false negative scores, using Pearson correlation tests. Results with a *p* < 0.05 were considered statistically significant. The feasibility of this proof-of-concept study was evaluated based on self-reported tolerability and test reliability scores. All statistical analyses were conducted using MedCalc (v.19.1.7, MedCalc Software, Ostend, Belgium).

## 3. Results

Twelve consecutive subjects (twenty-three eyes) were enrolled in December 2024. Subjects included seven females (58.3%) and five males (41.7%). The mean cohort age was 45 ± 15.2 years (range: 30–68). All subjects were Caucasian. While none of the subjects suffered from glaucoma, ophthalmic history included previous refractive surgery (17.4%, n = 4), amblyopia (17.4%, n = 4), esotropia (4.3%, n = 1), and high myopia (17.4%, n = 4). One patient was monophthalmic. Seven subjects (58.3%) wore their own spectacles within the headset.

Mean self-reported comfort score was high, at 8.75 out of 10 (range: 8–10), with some subjects taking the test in the dorsal decubitus position. In terms of acceptability, 11 subjects (91.7%) considered the device to be easy to use, and 100% responded they would accept repeating the test at home, of which 41.7% (n = 10) stated they would prefer to be supervised by a clinician. Overall, two subjects reported mild side effects, namely light asthenopia, epiphora, and periocular flushing. All side effects were mild and self-limited. Eleven subjects (91.7%) provided spontaneous positive subjective feedback, while one subject (8.3%) gave neutral feedback regarding their experience with VR-based VF testing. Positive comments frequently highlighted the practicality of the headset, particularly its allowance for flexible positioning, and six participants (50%) also noted the entertaining nature of the test. The neutral feedback acknowledged the engaging design but emphasized that the test still required focus and concentration to be completed properly. This feedback was expressed by the subject who had reported epiphora and periocular flushing. The most common positive terms used were “pleasant”, “practical”, “entertaining”, and “lightweighted” (n = 9, 8, 6, 3), while the neutral term used was “demanding” (n = 1; closest English translation).

The mean perceived duration of the test (187 s) correlated strongly with the mean actual duration (166 s; r = 0.76; *p*-value = 0.007). In all, 58.3% (n = 7) of the perceived durations were shorter than the actual test durations.

Mean false negative and false positive responses were 3.75% and 4.7%, respectively. Central fixation recorded by real-time eye tracking was maintained on average 73.23% of the time and showed a strong correlation with false negative responses (r = 0.75; *p* = 0.026).

## 4. Discussion

In this cohort, a large majority of subjects (91.7%, n = 11) considered the VR VF test to be easy to use and all of them would be willing to repeat VF tests at home if this was clinically needed. The mean self-reported comfort score was high (8.75/10), with only three subjects reporting mild, self-limited side effects. It has been reported that VR experiences may generate cybersickness, particularly when subjects are focused on their wider field of view [8]; however, the short duration and stable visual environment of the VF test seemed to alleviate this phenomenon in our cohort of participants. Interestingly, more than half the subjects (58.3%, n = 7) perceived a shorter duration of testing than the actual VF test duration. This might suggest that the comfort provided by the VR VF headset testing offered a less tiring and less stressful experience for the users compared to standard hospital or clinic-based monitoring. Although the size of the cohort did not allow for any formal regression analysis, there did not appear to be any correlation between subjects’ ages and their acceptance of the technology.

These findings align with the study by Hu et al., in which they reported that home VR VF was generally feasible, acceptable, and easy to use. [9] In addition, their cohort of glaucoma patients reported a heightened sense of security and insight about their condition. Self-reported results from their study reported similar levels of acceptability (94.4%) and ease of use (73.7%) to those described in the present study. Wroblewski and colleagues reported similar findings in terms of patient acceptability and usability of a VR headset for VF testing [10]. In their study, patients expressed a preference for the VR device over the standard Humphrey VF device for VF testing. Moreover, comparing clinical results across the two devices, they concluded on the reliability of VF measurements acquired via a VR headset, and suggested these devices may prove useful to a wide range of patients who may not normally be able to perform frequent in-office VF or use traditional devices.

In a recent systematic review, Hekmatjah et al. analyzed 14 studies comparing standard automated perimetry using devices, such as the Humphrey Field Analyzer and Octopus 900, with 10 different VR VF systems [11]. The authors concluded that VR VF shows promising evidence for its utility and feasibility in monitoring VFs in adults with glaucoma, though they emphasized the need for further validation, particularly regarding test–retest reliability and performance in varying disease severities. Other authors have examined different devices, concluding that other VR headsets, including the VisuALL S and Radius, offer superior comfort and flexibility compared to standard automated perimetry [12,13,14]. These studies often highlight the limitations of conventional testing, including the need for monocular occlusion and strict head positioning. By removing these constraints, and thanks to their portable and ergonomic design, VR systems are particularly suitable for elderly or mobility-impaired individuals. Patient-reported outcomes consistently reflect improved acceptability and reduced fatigue. In terms of reliability, VR-based VF testing has demonstrated strong correlations with conventional perimetry, with several studies reporting high agreement in mean deviation (MD) values and reduced test–retest variability, false positive rates, and fixation losses, including with the presently used SORS algorithm [5] Nevertheless, discrepancies have been noted in certain devices. For instance, the Smart System Virtual Reality (SSVR) device has been shown to overestimate nasal sensitivity and underestimate central sensitivity, underscoring the importance of validating new platforms through detailed pointwise sensitivity analyses and establishing clinical equivalence [15]. Beyond their portability, VR-based systems offer logistical advantages, such as the ability to test both eyes without external occlusion and to perform reliably under various ambient lighting conditions, reducing dependence on clinical infrastructure and personnel. Crucially, by enabling more frequent and accessible VF testing, VR perimetry may facilitate earlier detection of disease progression, particularly in the context of glaucoma monitoring [16].

The eye tracker allows a real-time control of the subject’s gaze direction through the entire examination. Although standard visual field devices are also equipped with gaze-tracking capability, the fixation is often checked using the blind spot location as a control. Continuous fixation rates have not yet been validated for VF testing, and, therefore, there is no reference as to what scores should constitute a reliability threshold. Nevertheless, in the studied device, the eye-tracker recorded a seemingly satisfactory fixation on average (73.23%), and individual eye-tracking scores showed a strong correlation with traditional reliability markers, such as false negative (FN) responses (r = 0.75). Considering that the reliability of the VF results is strongly correlated with the quality of the fixation gaze, the real-time eye-tracker provided by VR devices may prove to be a useful tool in monitoring patients and confirming the reliability of VF results. From a practical point of view, the immersive nature of the VR device may also contribute to improving test reliability by controlling the testing environment and reducing the effects of external light sources and distractions.

A few studies have observed the benefits of home testing using VR headsets, notably via an increase in test frequency [1,17,18,19]. However, arranging long-term home testing as a standard for large number of patients may constitute a logistical challenge, and some patients appear to value direct medical supervision and immediate feedback from their physicians. Nevertheless, VR headsets may find their true value in more traditional settings, in hospitals and ophthalmology offices, where an improvement in patient comfort and operational aspects may lead to better detections of glaucoma progression through higher test frequencies [16]. Indeed, although Glen et al. reported that despite patients’ general dislike of visual field testing, they would be happy to perform as many tests as clinically indicated [20], we have observed through our practice and experience that many patients attempt to bargain in order to avoid or reduce the frequency of VF tests. Offering patients a more comfortable alternative to traditional VF testing may remove one of the barriers to meeting guidelines in terms of VF frequencies.

Beyond these advantages for most glaucoma patients, VR headsets may also improve accessibility to VF testing. Indeed, a small proportion of glaucoma patients are unable to perform regular testing due to positioning limitations, such as spinal or neurological conditions, including bedbound patients. In the present study, some subjects found the positioning for a slit-lamp examination difficult and instead performed their VF test in the dorsal decubitus position. This may offer a solution for bedbound patients to improve their access to comprehensive glaucoma care.

The present study has significant limitations. Its focus on patient comfort and acceptability means that the present study did not examine the test algorithm itself or its comparability with traditional devices. The small cohort of young, Caucasian, non-glaucomatous subjects may not be representative of a typically older group of glaucoma patients and precluded the use of more advanced analyses, such as regression analyses. Given the small number of subjects, this exploratory study relies on correlation tests and percentages, focusing on the reported subjective feedback. Nevertheless, this is in keeping with the numbers used in other acceptability studies, and ad hoc power analysis suggests a risk of Type I error of approximately 10% and a Type II error of 5%, which provides some reassurance regarding the reliability of the observed comfort ratings. Similarly, a proportion of the cohort were naïve to visual field testing. Although Chew et al. reported that anxiety tends to decrease with increased test experience [6], it may also be speculated that the low-stakes experimental setting could have made the test less stressful and, therefore, more comfortable. Finally, the absence of a comparison with a traditional VF device restricts the generalization of the present observations.

In conclusion, the present study suggested that VF testing using a VR headset is well tolerated and accepted, with high levels of self-reported comfort and only a few limited mild side effects. While all subjects welcomed the opportunity to perform clinical tests from home, over a third expressed a preference for supervision. More research is warranted to determine the value of home testing in the context of glaucoma care, and to confirm the acceptability of VR VF more specifically in a cohort of elderly and glaucomatous patients. Nevertheless, VR VF monitoring either at home or in clinical settings might be a promising solution for a more comfortable, accessible, and personalized approach in glaucoma care, notably in patients whose general health may preclude regular monitoring. Additionally, real-time eye tracking correlated well with traditional reliability markers, suggesting potential clinical value.

## Data Availability

The original contributions presented in this study are included in the article. Further inquiries can be directed to the corresponding author.
